# Saliva is Comparable to Nasopharyngeal Swabs for Molecular Detection of SARS-CoV-2

**DOI:** 10.1128/spectrum.00162-21

**Published:** 2021-08-18

**Authors:** Cody Callahan, Sarah Ditelberg, Sanjucta Dutta, Nancy Littlehale, Annie Cheng, Kristin Kupczewski, Danielle McVay, Stefan Riedel, James E. Kirby, Ramy Arnaout

**Affiliations:** a Department of Radiology, Beth Israel Deaconess Medical Center, Boston, Massachusetts, USA; b Department of Pathology, Beth Israel Deaconess Medical Center, Boston, Massachusetts, USA; c Division of Clinical Informatics, Department of Medicine, Beth Israel Deaconess Medical Center, Boston, Massachusetts, USA; d Beth Israel Deaconess Medical Center, Boston, Massachusetts, USA; e Harvard Medical School, Boston, Massachusetts, USA; University of Cincinnati

**Keywords:** SARS-CoV-2, COVID-19, saliva, NP swab, limit of detection

## Abstract

The continued need for molecular testing for severe acute respiratory syndrome coronavirus 2 (SARS-CoV-2) and the potential for self-collected saliva as an alternative to nasopharyngeal (NP) swabs for sample acquisition led us to compare saliva to NP swabs in an outpatient setting without restrictions to avoid food, drink, smoking, or tooth-brushing. A total of 385 pairs of NP and saliva specimens were obtained, the majority from individuals presenting for initial evaluation, and were tested on two high-sensitivity reverse transcriptase PCR (RT-PCR) platforms, the Abbott m2000 and Abbott Alinity m (both with limits of detection [LoD] of 100 copies of viral RNA/ml). Concordance between saliva and NP swabs was excellent overall (Cohen’s κ = 0.93) for both initial and follow-up testing, for both platforms, and for specimens treated with guanidinium transport medium as preservative as well as for untreated saliva (κ = 0.88 to 0.95). Viral loads were on average 16× higher in NP specimens than saliva specimens, suggesting that only the relatively small fraction of outpatients (∼8% in this study) who present with very low viral loads (<1,600 copies/ml from NP swabs) would be missed by testing saliva instead of NP swabs when using sensitive testing platforms. Special attention was necessary to ensure leak-resistant specimen collection and transport. The advantages of self-collection of saliva, without behavioral restrictions, will likely outweigh a minor potential decrease in clinical sensitivity in individuals less likely to pose an infectious risk to others for many real-world scenarios, especially for initial testing.

**IMPORTANCE** In general, the most accurate COVID-19 testing is hands-on and uncomfortable, requiring trained staff and a “brain-tickling” nasopharyngeal swab. Saliva would be much easier on both fronts, since patients could collect it themselves, and it is after all just spit. However, despite much interest, it remains unclear how well saliva performs in real-world settings when just using it in place of an NP swab without elaborate or cumbersome restrictions about not eating/drinking before testing, etc. Also, almost all studies of COVID-19 testing, whether of NP swabs, saliva, or otherwise, have been restricted to reporting results in the abstruse units of “*C_T_* values,” which only mean something in the context of a specific assay and testing platform. Here, we compared saliva versus NP swabs in a real-world setting without restriction and report all results in natural units—the amount of virus being shed—showing that saliva is essentially just as good as NP swabs.

## INTRODUCTION

The currently accepted gold-standard method for diagnosing severe acute respiratory syndrome coronavirus 2 (SARS-CoV-2) infection is reverse transcriptase PCR (RT-PCR) on nasopharyngeal (NP) secretions collected using an NP swab. This choice is consistent with and was in part based on experience with other respiratory pathogens, such as influenza viruses. However, the nasopharynx has the drawback of requiring experienced health care staff to perform sample collection, and such personnel are in chronic short supply. Also, requiring potentially infectious patients to congregate at a collection site is not ideal, as it may inadvertently lead to iatrogenic infection. These suboptimalities have led to a number of investigations into whether and under what clinical circumstances alternative specimen types might be able to substitute for NP swabs (1–12). Nasal secretions have the advantage of being able to be self-collected by swabbing the anterior nares; however, nasal secretions have been shown to be less sensitive than NP secretions for viral loads below ∼1,000 copies/ml (5, 6, 13), and nasal secretions still require a swab for collection (swabs have been in intermittently short supply over the course of the pandemic) (14).

Saliva has the advantage of requiring neither trained personnel nor a swab, making it an attractive alternative to both NP and/or nasal swab testing. Saliva can be self-collected using only a sterile container, making it amenable to at-home/off-site collection. Sensitive detection of respiratory viruses from saliva had been shown prior to the pandemic (15, 16). Consequently, saliva has been the subject of a number of studies in the context of the pandemic; however, these have reached varying conclusions regarding the suitability of saliva as an alternative to NP swabs, with some studies showing complete concordance and others only moderate agreement (9). Known and potential reasons for these differences include timing/acuity of patients’ clinical presentation, storage/processing conditions of collected samples, and sensitivity of the respective testing platforms used for detection of SARS-CoV-2. To address these issues, we compared saliva to NP swabs in 385 subjects across two testing platforms (Abbott m2000 and Abbott Alinity m) and two sample-processing approaches (untreated/“neat” and treated with guanidinium as a preservative) for individuals presenting for initial presentation versus follow-up testing.

## RESULTS

### Study design and overall observations.

**FIG 1:**
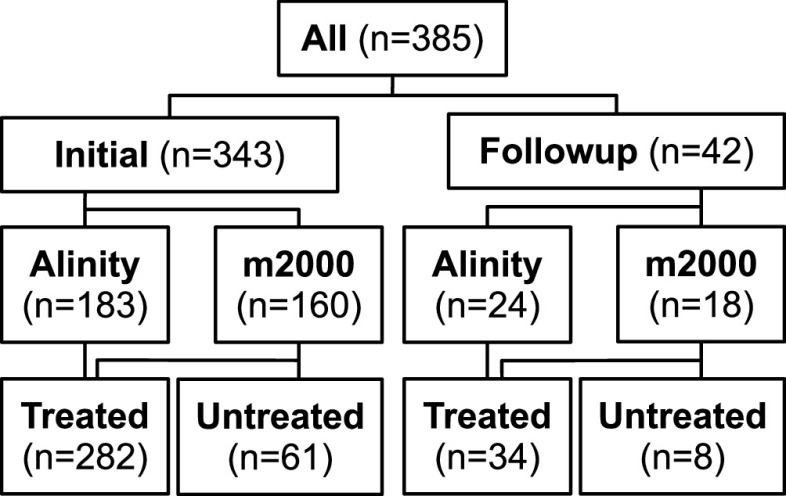
Numbers of participants in each study arm.

Figure 1 depicts the design of this study of adults being tested by RT-PCR for infection with SARS-CoV-2 and the numbers of participants tested in each arm. Participants waiting for NP sample collection were asked to spit into a sterile urine cup clearly marked with a 3-ml fill line until the line was reached. Participants were informed that this would likely require spitting more than once, encouraged to persist despite any embarrassment or distaste/mild disgust, and instructed to thread the lids carefully and close them tightly to avoid leaking. Study staff retightened cups as necessary. Initially, saliva specimens were placed in the same bag as the NP specimen, but after frequent leaks through the cup threads (∼40%, which required hood decontamination and NP recollection), we switched to the gasketed cups discussed in Materials and Methods, bagged saliva specimens individually, and placed these bags in an outer bag with the NP specimen for transport (≤20% leaks, which only rarely required recollection). These experiences highlight the importance of the choice of saliva cup (e.g., ones with a gasket and a thread pitch that avoids slipping, misalignment, or saliva leaking into the threads), and of evaluating cups before use.

### Limit of detection of SARS-CoV-2 in saliva on Abbott m2000 and Alinity m.

**FIG 2:**
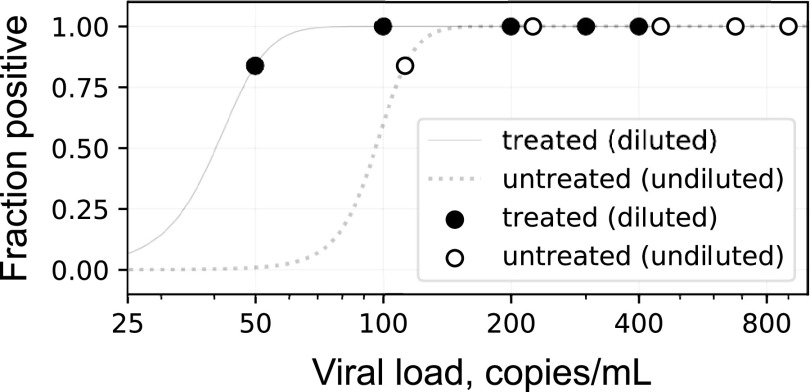
LoD for treated and untreated saliva on Abbott m2000 and Alinity m platforms. Lowest two datapoints with 24 replicates each; others in triplicate.

In spiking experiments, the lowest concentration level with observed positive rates of ≥95% was 100 copies/ml in treated saliva and 225 copies/ml in untreated saliva. Logistic regression estimated limits of detection (LoDs) of 45 copies/ml in treated and 104 copies/ml in untreated saliva (76 copies/ml combined), which is statistically comparable to the previously validated LoD for NP swabs on these platforms, 100 copies/ml (Fig. 2). Note that treatment results in a roughly 2-fold dilution of the sample.

### Stability of SARS-CoV-2 in saliva over time.

**FIG 3:**
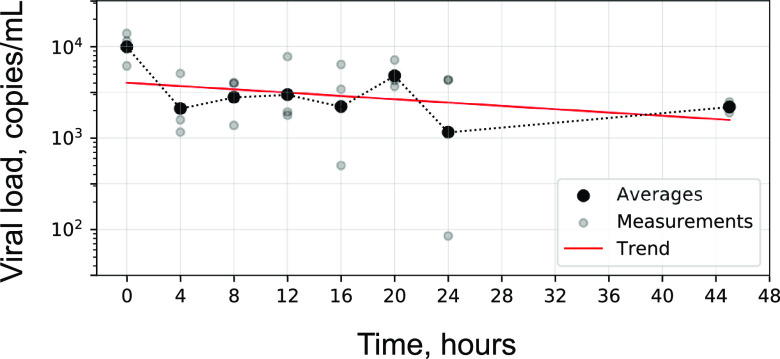
Stability of replication-incompetent SARS-CoV-2 surrogate virus in untreated saliva samples over time. Gray, triplicate measurements; black, geometric means; red, exponential decay fit.

Replication-incompetent, enveloped, positive single-stranded RNA Sindbis virus into which SARS-CoV-2 genomic material was cloned as a noninfectious SARS-CoV-2 surrogate was stable in untreated saliva at room temperature, with concentration falling by ∼3 fold within the first 4 h but then staying stable at that level through 2 days (Fig. 3). Saliva treated with the preservative guanidinium isothiocyanate (GITC) was stable for at least 24 h. Together, these findings support the viability of workflows in which, as a limiting timeline, saliva is collected and transported “neat” and treated with GITC within 2 days for subsequent testing.

### Comparison of saliva to NP swabs.

**FIG 4:**
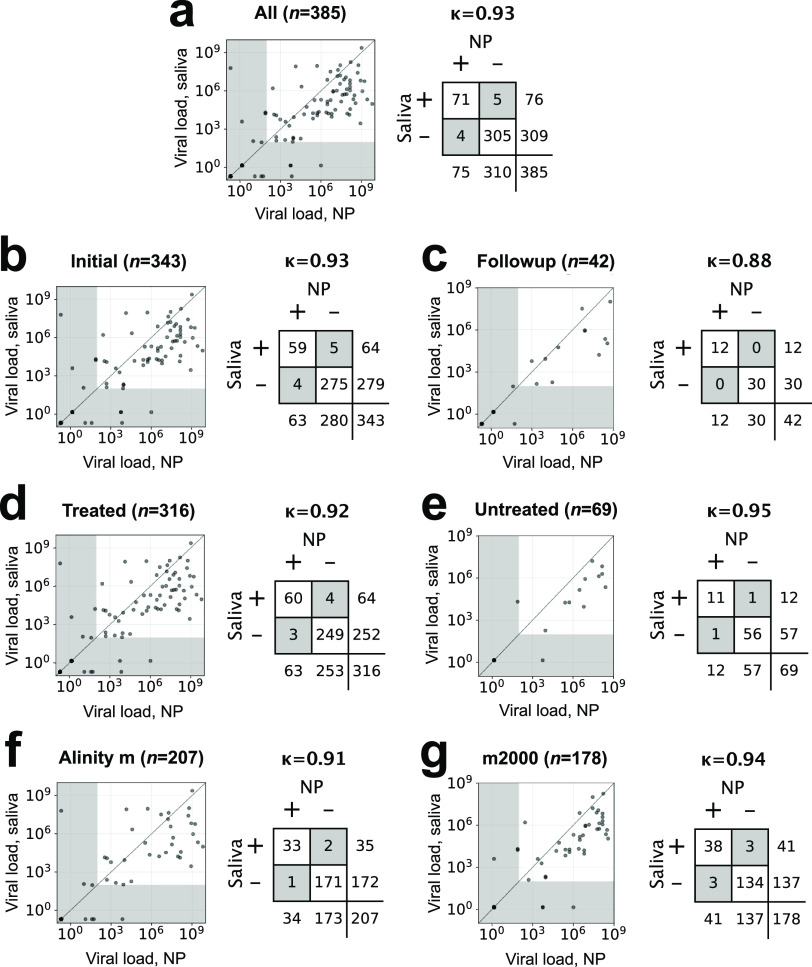
Viral load in saliva versus NP swab samples, Cohen’s kappa (κ) concordance values, and contingency tables for overall study (a), subjects presenting for initial presentation (within 5 days of first COVID-19 RT-PCR test) (b), subjects presenting for follow-up testing (c), samples treated with GITC transport buffer as a preservative after receipt at the central laboratory (d), untreated samples (e), samples run on the Alinity m platform (f), and samples run on the m2000 (g). Diagonal lines in scatterplots, 1:1. Gray shaded areas in the scatterplots are below the LoD (100 copies/ml). Gray shaded cells in the contingency tables highlight discordant results.

**FIG 5:**
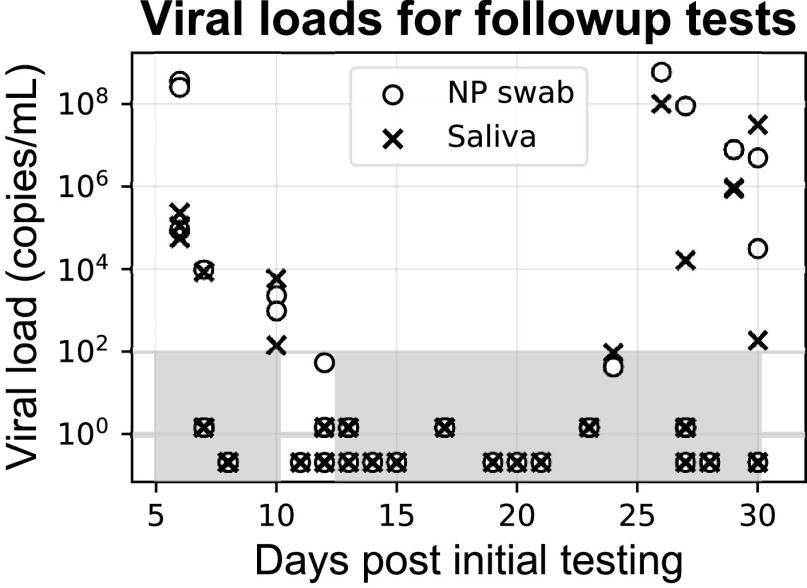
Sample processing times ranked by increasing order of total time. Sample-to-login time is the time between sample acquisition and login at the central laboratory. Login-to-processing time is the time between login and arrival of the specimen in the molecular laboratory when GITC treatment and RT-PCR testing are performed.

Overall, saliva results were highly concordant (Cohen’s κ = 0.93) with results from paired NP swabs obtained from participants in a medium-to-high prevalence population (∼20% positivity) who presented for screening and follow-up, as tested on two sensitive RT-PCR platforms, the Abbott Alinity m and Abbott m2000 (LoD, 100 copies/ml; recall the LoD is the limit below which positivity cannot be ascertained with ≥95% confidence) (Fig. 4). Using the LoD as the positivity cutoff, there were only nine discordant results among 385 paired samples, and these were evenly balanced (4 NP-swab-positive/saliva-negative samples versus 5 saliva-positive/NP-swab-negative samples). Despite the high concordance, (geometric) mean viral load in NP samples was ∼16 times as high as that in saliva samples (3.1 million versus 200,000 copies/ml, respectively; Wilcoxon *P* value for difference of saliva versus NP viral load distributions, 2 × 10^−6^). These trends were robust across all subgroups analyzed, whether by initial testing (*n *= 343; κ = 0.93) or follow-up testing (*n *= 42; κ = 0.88), whether samples were treated with GITC as soon as they reached the laboratory (Fig. 5) (*n *= 316; κ = 0.92) or not (*n* = 69; κ = 0.95), and whether they were run on the Alinity m (*n* = 207; κ = 0.91) or m2000 (*n* = 178; κ = 0.94). Notably, mean viral load for NP swab samples was 13 times as high as that for saliva samples treated with GITC versus 40 times as high as that for untreated saliva samples. Mean viral load was 2× to 3× as high that for participants presenting for their initial COVID-19 test versus follow-up testing (3.7 million versus 1.5 million copies/ml, respectively, as measured on NP samples, and 240,000 versus 81,000 copies/ml, respectively, as measured on saliva samples) (see Fig. S1 in the supplemental material).

We found nothing unusual in the 5 NP-negative/saliva-positive discordant samples or the 4 NP-positive/saliva-negative samples (Fig. 4a, gray regions >100 copies/ml along the *x* or *y* axis). Two of these were very nearly concordant positive, with >10^4^ copies/ml in saliva and just under 100 copies/ml by NP sample. A third sample was very nearly concordant negative, with viral load just above 100 copies/ml in saliva but ∼10 copies/ml in NP sample. The remaining six were more highly discordant; absent further information, we consider these random errors. The general symmetry of these discordant results is consistent with the conclusion that NP and saliva are generally comparable.

## DISCUSSION

While saliva testing has shown promise for saliva as a suitable specimen for detection of several viral respiratory pathogens (8–10, 15), NP swabs remain the gold standard in most clinical settings, requiring well-trained health care staff to obtain them. The demands of the COVID-19 pandemic have led to a number of investigations of saliva for molecular detection of SARS-CoV-2 in a wide range of clinical and nonclinical settings and with a variety of patient instructions and processing steps, with the overall conclusion that saliva is comparable to but slightly inferior to NP swabs (17). Some studies have instructed patients to provide morning specimens or to abstain from eating/drinking/smoking before providing a specimen, constraints that complicate collection. We sought to test how well saliva testing in a real-world outpatient setting without any such constraints would perform, with the only special handling step being adding a preservative, in the form of GITC transport buffer, once the specimen arrived from the outpatient site to our hospital’s central laboratory for testing.

The overall results in our current study were consistent with previous work (17), with very high concordance between saliva and NP swabs under all scenarios tested. A minor but surprisingly important observation was the importance of leak-resistant collection cups, as a substantial number of collected specimens were unusable due to leaks, despite proper instruction for sample collection. The leak-resistant cups that we adopted mitigated leaking somewhat, as they incorporated a soft gasket-like seal inside the lid, with a thread length to pitch ratio of ∼3. Cups using a more deformable gasket or larger ratio may further reduce sample leakage. Nevertheless, our experience demonstrates that simple and readily available specimen collection cups can be repurposed for saliva collection for respiratory virus testing. Usefully, we observed high concordance without any restrictions on participant activities (eating/drinking/smoking/tooth brushing; the color and heterogeneity of several saliva samples was highly suggestive of these activities) without asking participants to rinse, cough, or retch and without requiring specimens to be obtained in the morning (indeed most of the samples were collected in the early afternoon). While it cannot be ruled out that these additional requirements might improve performance (∼5% improvement in concordance in meta-analysis [20]), the results of the present study suggest that the procedural hurdles of obtaining specimens with these constraints likely outweigh any modest incremental benefit.

Furthermore, we demonstrated that SARS-CoV-2 viral material was stable in GITC-treated saliva for at least 24 h. The time between sample collection and addition of GITC never exceeded 12 h. Treatment was performed in the laboratory and not earlier, at the collection site, for simplicity of workflow. We chose not to include the preservative in the specimen collection cups for concern over potential exposure of participants to a caustic substance. The median time between specimen collection and addition of GITC was approximately 6 h (Fig. 5); however, we demonstrated that viral material is fairly stable in saliva at room temperature without preservative, retaining approximately a third of viral load at 24 h and 45 h (Fig. 3, red), allowing room temperature transport and login times of over a day. Consistent with these stability studies, untreated saliva samples showed indistinguishably high concordance with NP swabs compared to that of GITC-treated saliva samples (Fig. 4d and e), with the mean viral load in untreated samples approximately 3× lower (also consistent with stability studies).

Despite the high concordance overall (κ = 0.93) and for all subgroup analyses (κ = 0.88 to 0.95), including for participants presenting for follow-up as well as during initial presentation, the on average 13× higher viral load detected by NP swabs versus paired GITC-treated saliva specimens deserves mention. Provided that viral load (as measured by NP swab) is greater than 13 times the platform’s LoD (100 copies/ml in our study), saliva treated with GITC within 24 h of collection is highly likely to detect infection. However, viral loads less than this threshold (1,300 copies/ml) may be missed. In our study, approximately 92% of initial presentations were above this threshold. The analogous ratio for untreated saliva was 40×, corresponding to a threshold of 4,000 copies/ml; approximately 90% of initial presentations were above this threshold. We conclude that saliva can be fairly expected to detect over 90% of initial SARS-CoV-2 infections in an outpatient setting. To the extent that more severe disease correlates with higher viral loads, performance in inpatient settings is likely to be even higher. Furthermore, a detection level in saliva that would correspond to 4,000 copies/ml in NP swab samples is likely to capture those who are infectious and is also likely significantly below the limit of detection of currently marketed SARS-CoV-2 antigen tests (18).

In conclusion, we have demonstrated that saliva is comparable to NP swab collection for molecular detection of SARS-CoV-2 in an outpatient setting, with the disadvantage of slightly lower viral loads. This loss in sensitivity must be weighed against the ease of self-collection. However, we have demonstrated that use of saliva for SARS-CoV-2 molecular detection is both feasible and practical, given suitable specimen collection containers and transport and processing protocols.

## MATERIALS AND METHODS

### Institutional review.

This study was reviewed and approved by institutional review board (IRB) at the Beth Israel Deaconess Medical Center (BIDMC) (IRB protocol no. 2020P000769).

### Trial design.

This was a multi-arm study with the following parameters: initial versus follow up presentation, two different testing platforms (Abbott m2000 and Abbott Alinity m), and two different sample-processing pipelines for the saliva samples (unadulterated/“neat” saliva versus preserved [“treated”] with guanidinium isothiocyanate [GITC], i.e., Abbott multi-Collect transport media, part of the Abbott multi-Collect specimen collection kit, catalog no. 09K12-004; Abbott Laboratories, Abbott Park, IL) (see Fig. 1).

### Trial participants and sample collection.

Informed consent was obtained from English-speaking adults presenting for either initial or follow-up testing for COVID-19 at BIDMC and Beth Israel Deaconess Chelsea drive-through collection sites. Participants included a mix of symptomatic and asymptomatic patients (e.g., including some preparing for holiday travel).

### (i) Saliva specimens.

While waiting in line for NP testing, each participant was given a sterile sample collection cup (VWR International; part no. 76299-868) and asked to spit/drool to a clearly labeled 3 ml fill line. No exclusions were made on the basis of recent consumption of food or drink or of smoking, and participants were not asked to rinse the mouth or perform any other preparation ahead of sample collection. They were informed that 3 ml is a large amount of saliva and may take several minutes/deposits to achieve and that thinking of a favorite food may help trigger salivation. They were told to close the cup tightly when finished and to hand it to the health care worker who would be acquiring the NP specimen.

### (ii) NP specimens.

NP specimens were collected per standard protocol. Briefly, the NP swab was inserted into the nasopharynx and rotated for 10 s, removed, and placed in a vial containing viral transport medium (19).

### (iii) Transport.

Saliva specimens were individually bagged and transported together with the NP specimen via courier to BIDMC’s central laboratory for testing. Courier frequency was every 1 to 2.5 h. The courier’s maximum transport time between the collection site and the laboratory was approximately 20 min. Specimens were transported at room temperature. At the BIDMC Pathology central laboratory, specimens were logged in and processed for testing as described below.

### Sample processing.

For GITC-treated saliva samples, 1 ml of saliva was added to the multi-Collect tube that contains 1.25 ml GITC buffer (“treated”). For untreated saliva samples, 1 ml was used “neat” (i.e., without addition of GITC). Samples with <1 ml were excluded.

Treated/untreated samples and a 1-ml aliquot of NP sample from the same participant were briefly vortexed (2 to 5 s) and amplified using Abbott m2000 RealTime SARS-CoV-2 assay on an Abbott m2000 RealTime system (“m2000”) or the Abbott Alinity m system (“Alinity”). Untreated specimens were run concurrently with the NP sample. After determination of stability of treated samples (see Stability Testing below), treated specimens were stored at 4°C until the paired NP sample had resulted (never longer than 14 h; 98% were within 10 h) (see Fig. 5). If the NP sample was positive, the GITC sample was run alongside an additional 1-ml aliquot from the NP sample. Categorical (positive versus negative), *C_T_*, and internal control (IC) values were recorded. Cutoff for positive was a *C_T_* value of 31.5 on the m2000 and 42 on the Alinity m.

Initially, all samples were processed regardless of whether the NP result was positive or negative; for these, negatives outnumbered positives, which is consistent with the COVID-19 positivity rate in the population. After 12 positive untreated specimens, the untreated arm was abandoned to prioritize clinical bandwidth. After several days of collection, resulting in a majority of negatives, only positives were included to minimize clinical burden on laboratory staff.

### Limits of detection. (i) NP swabs.

The limit of detection (LoD) for SARS-CoV-2 from NP swabs was validated at 100 copies/ml on both the m2000 and Alinity m platforms as described previously (18).

### (ii) Saliva.

To determine the LoD for saliva, a (noninfectious) recombinant, enveloped, positive single-stranded RNA virus containing SARS-CoV-2 RNA (AccuPlex COVID-19; SeraCare, Milford, MA, USA; 1.3 × 10^7^ copies/ml as determined by digital PCR) was initially diluted into RNA storage solution purchased from Invitrogen (AM7001; Thermo Fisher Scientific, Waltham, MA, USA) to make a positive stock. The positive stock was then spiked to target concentrations in pooled negative saliva prepared with purchased leftover remnant deidentified specimens. A portion of these spiked “neat” or “untreated” samples were combined with transport medium using a saliva/GITC volume ratio of 1:1.25; these are “treated” samples. Tested concentrations were 900, 675, 450, 225, and 112.5 copies/ml for untreated samples and 400, 300, 200, 100, and 50 copies/ml for treated samples. The initial LoD was determined by testing targeted levels in replicates of 3; the final LoD was confirmed by testing in 21 additional replicates of the lowest two dilutions.

### Stability testing.

Saliva specimens were collected as described above and stored at 4°C pending results of the paired NP specimen. Twenty NP confirmed negative saliva specimens were pooled. The pooled sample was spiked to a final concentration of 11,215 copies/ml of SeraCare material and aliquoted into 24× 1.1-ml replicates. Starting immediately (the zero-hour time point), each of three replicates was treated with 1.2 ml GITC and promptly run on the Alinity m platform per standard protocol. This was repeated at 4, 8, 12, 16, 20, 24, and 45 h. Replicates were stored at room temperature between time points. Categorical, *C_T_*, and IC values were recorded.

### Sample-processing times.

NP specimens were tracked using BIDMC’s clinical data system, with the following recorded: order creation time as a surrogate for sample collection time (±15 min), lab-control time (sample login at BIDMC), login time at BIDMC’s molecular diagnostics laboratory, archive time, and result time.

### Statistical analyses.

For concordance testing, RT-PCR results were considered categorically as either positive or negative; testing agreement was assessed by Cohen's kappa (*κ*) (20). Conversion to viral load was performed as described previously (18) using the definition of exponential growth with variable efficiency (21, 22), with efficiency measured from time series of fluorescence intensity versus cycle number. “Initial testing” was defined as testing of a saliva sample collected fewer than 5 days after the first COVID-19 RT-PCR test. “Follow-up testing” was defined as testing occurring thereafter for follow up of previously positive results or suspicion of past exposure. Leaking samples and internal-control failures were omitted from analysis.

### Significance testing.

We tested whether *C_T_* values for a given subset of saliva samples differed from the *C_T_* values for the paired NP swabs (the controls) using Wilcoxon’s signed rank test. This tested the null hypothesis that values for controls and prototypes are drawn from the same underlying distribution. The Benjamini-Hochberg approach (23) was used to control the false-discovery rate (FDR) at 1%.

### Software.

We used Python (v3.6–3.8) and its NumPy, SciPy, Matplotlib, Pandas, and ct2vl libraries for the above analyses and related visualizations.

### Data availability.

Deidentified data is presented in Supplemental File S1. Supplemental data can be found at https://github.com/rarnaout/Covid_diagnostics.
