# Supplementary figures and images for: Saliva is Comparable to Nasopharyngeal Swabs for Molecular Detection of SARS-CoV-2
Source: Microbiol Spectr. 2021 Aug 18;9(1):10.1128/spectrum.00162-21. doi: 10.1128/spectrum.00162-21 (PMC8552668; doi:10.1128/spectrum.00162-21)

Fig. S1. Viral loads for follow-up tests. See main text for details.

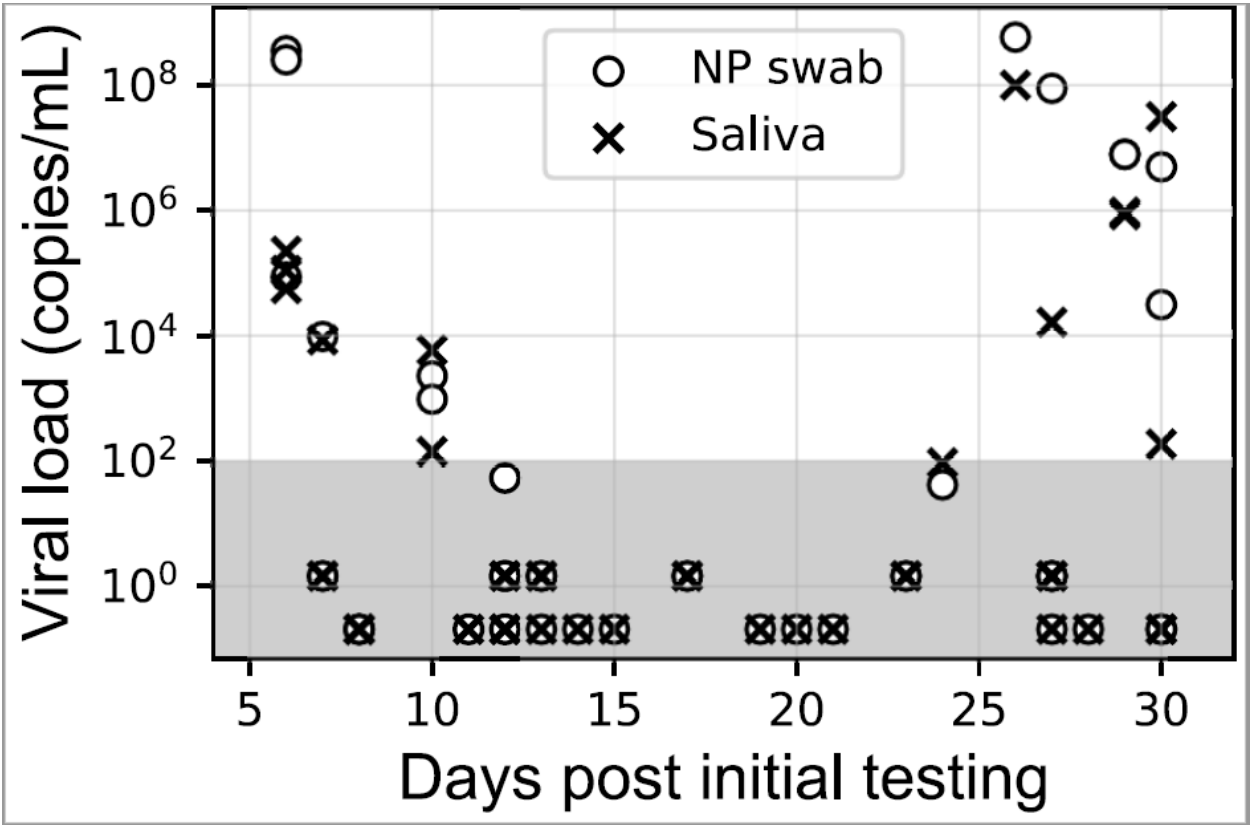

Supplement: SUPPLEMENTAL FILE 1 — Supplemental material. Download SPECTRUM00162-21_Supp_1_seq15.pdf, PDF file, 0.1 MB [file spectrum00162-21_supp_1_seq15.pdf]
